# External memory BWT and LCP computation for sequence collections with applications

**DOI:** 10.1186/s13015-019-0140-0

**Published:** 2019-03-08

**Authors:** Lavinia Egidi, Felipe A. Louza, Giovanni Manzini, Guilherme P. Telles

**Affiliations:** 1 DiSIT, University of Eastern Piedmont, Viale Michel, 11, 15121 Alessandria, Italy; 2 Department of Computing and Mathematics, University of São Paulo, Av. Bandeirantes, 3900, 14040-901 Ribeirão Preto, Brazil; 3 IIT CNR, Via Moruzzi, 1, 56124 Pisa, Italy; 4 Institute of Computing, University of Campinas, Av. Albert Einstein, 1251, 13083-852 Campinas, Brazil

**Keywords:** Burrows–Wheeler Transform, Longest common prefix array, Maximal repeats, All pairs suffix–prefix overlaps, Succinct de Bruijn graph, External memory algorithms

## Abstract

**Background:**

Sequencing technologies produce larger and larger collections of biosequences that have to be stored in compressed indices supporting fast search operations. Many compressed indices are based on the Burrows–Wheeler Transform (BWT) and the longest common prefix (LCP) array. Because of the sheer size of the input it is important to build these data structures in external memory and time using in the best possible way the available RAM.

**Results:**

We propose a space-efficient algorithm to compute the BWT and LCP array for a collection of sequences in the external or semi-external memory setting. Our algorithm splits the input collection into subcollections sufficiently small that it can compute their BWT in RAM using an optimal linear time algorithm. Next, it merges the partial BWTs in external or semi-external memory and in the process it also computes the LCP values. Our algorithm can be modified to output two additional arrays that, combined with the BWT and LCP array, provide simple, scan-based, external memory algorithms for three well known problems in bioinformatics: the computation of maximal repeats, the all pairs suffix–prefix overlaps, and the construction of succinct de Bruijn graphs.

**Conclusions:**

We prove that our algorithm performs ${\mathcal {O}}(n\, \mathsf {maxlcp})$ sequential I/Os, where *n* is the total length of the collection and $\mathsf {maxlcp}$ is the maximum LCP value. The experimental results show that our algorithm is only slightly slower than the state of the art for short sequences but it is up to 40 times faster for longer sequences or when the available RAM is at least equal to the size of the input.

## Introduction

A fundamental problem in bioinformatics is the ability to efficiently search into the billions of DNA sequences produced by NGS studies. The Burrows Wheeler transform (BWT) [1] is a well known structure which is the starting point for the construction of compressed indices for collections of sequences [2]. The BWT is often complemented with the longest common prefix (LCP) array [3] since the latter makes it possible to efficiently emulate Suffix Tree algorithms [4, 5]. The construction of such data structures is a challenging problem. Although the final outcome is a *compressed* index, construction algorithms can be memory hungry and the necessity of developing *lightweight* algorithms was recognized since the very beginning of the field [6–8]. In lightweight algorithms it is assumed that the input and the output fit in main memory but the amount of additional working memory is sublinear with the size of the input.

When even lightweight algorithms do not fit in RAM, one has to resort to external or semi-external memory construction algorithms (see [9–14] and references therein). In the external memory model [15] it is assumed that the main memory grows at most polylogarithmically with the size of the input. In the semi-external model the main memory can grow linearly with the size of the input but part of the working data has to reside on disk. In both models the complexity of the algorithms is usually measured in terms of disk I/Os, since data transfer is much slower than CPU operations.

The space efficient computation of the BWT in main memory for a single sequence is well studied, and remarkable advances have been recently obtained [16, 17]. Unfortunately, for external memory computation the situation is less satisfactory. For collections of sequences, the first external memory algorithm is the BCR algorithm described in [18] that computes the multi-string BWT for a collection of total size *n*, performing a number of sequential I/Os proportional to *nK*, where *K* is the length of the longest sequence in the collection. This approach is clearly not competitive when the sequences have non-homogeneous lengths, and it is far from the theoretical optimal even for sequences of equal length. Nevertheless, the simplicity of the algorithm makes it very effective for collections of relatively short sequences, and it has become the reference tool for this problem. This approach was later extended [19] to compute also the LCP values with the same asymptotic number of I/Os. When computing also the LCP values, or when the input strings have different lengths, the algorithm uses ${\mathcal {O}}(m)$ words of RAM, where *m* is the number of input sequences.

In this paper, we present a new space-efficient algorithm for the computation of the BWT and LCP array for a collection of sequences in external or semi-external memory. Our algorithm takes the amount of available RAM as an input parameter, and tries to make the best use of it by splitting the input into subcollections sufficiently small so that it can compute their BWT in internal memory using an optimal linear time algorithm. Next, it merges the partial BWTs in external or semi-external memory and in the process it also computes the LCP values. Since the LCP values are computed in a non-standard order, the algorithm is completed by an external memory mergesort procedure that computes the final LCP array. We show that our algorithm performs a number of sequential I/Os between ${\mathcal {O}}(n\, \mathsf {avelcp})$ and ${\mathcal {O}}(n\, \mathsf {maxlcp})$, where $\mathsf {avelcp}$ and $\mathsf {maxlcp}$ are respectively the average and the maximum longest common prefix of the input sequences. To our knowledge, the only other known external memory algorithm for computing the BWT and LCP arrays of a collection of sequences is bwt-lcp-em, recently proposed in [20] that performs ${\mathcal {O}}(n\, \mathsf {maxlcp})$ sequential I/Os and uses ${\mathcal {O}}(m+K)$ words of RAM, where *K* is the fixed string length.

In “Related approaches” section we compare our approach with the ideas behind these previous works, and in “Experiments” section we compare their performance in practice. The experimental results show that BCR is the fastest algorithm for relatively short sequences while our algorithm is the fastest when the average LCP of the collection is relatively small compared to the lengths of the sequences. Both our algorithm and BCR appear to be faster than the available implementation of bwt-lcp-em, which is however only a prototype implementation with some limitations on the admissible inputs.

Another contribution of the paper, which follows from our first result, is the design of simple external memory algorithms for three well known problems related to genomic sequences, namely: (i) the computation of maximal repeats [21, 22], (ii) the computation of the all pairs suffix–prefix overlaps [23–25], and (iii) the construction of succinct de Bruijn graphs [26–28]. Our external memory algorithms for these problems are derived from known internal memory algorithms, but they process the input data in a single sequential scan. In addition, for the problem of computing the all pairs suffix–prefix, we go beyond the recent solutions that compute *all* the overlaps [24, 25, 29, 30], and we compute only the overlaps above a certain length, still spending constant time per reported overlap. Our algorithms take as input the BWT and LCP array, together with two additional arrays that our BWT construction algorithm can compute without any asymptotic slowdown.

Since problems on genomic sequences often involve huge datasets, it is certainly important to provide efficient external memory algorithms for the three problems described above. To our knowledge, only for the all pairs suffix–prefix problem there exists an external memory algorithm, namely the algorithm [30, Algorithm 2] that computes all the overlaps given the BWT, LCP, and Generalized Suffix Array of the input collection.

## Background

Let ${\mathsf {s}}_{}[1,n]$ denote a string of length *n* over an alphabet $\Sigma$ of constant size $\sigma$. As usual, we assume ${\mathsf {s}}_{}[n]$ is a special symbol (end-marker) not appearing elsewhere in ${\mathsf {s}}_{}$ and lexicographically smaller than any other symbol. We write ${\mathsf {s}}_{}[i,j]$ to denote the substring ${\mathsf {s}}_{}[i] {\mathsf {s}}_{}[i+1] \cdots {\mathsf {s}}_{}[j]$. If $j\ge n$ we assume ${\mathsf {s}}_{}[i,j] = {\mathsf {s}}_{}[i,n]$. If $i>j$ or $i > n$ then ${\mathsf {s}}_{}[i,j]$ is the empty string. Given two strings ${\mathsf {s}}_{1}$ and ${\mathsf {s}}_{2}$ we write ${\mathsf {s}}_{1} \preceq {\mathsf {s}}_{2}$ (${\mathsf {s}}_{1} \prec {\mathsf {s}}_{2}$) to denote that ${\mathsf {s}}_{1}$ is lexicographically (strictly) smaller than ${\mathsf {s}}_{2}$. We denote by $\mathsf {LCP}({\mathsf {s}}_{1},{\mathsf {s}}_{2})$ the length of the longest common prefix between ${\mathsf {s}}_{1}$ and ${\mathsf {s}}_{2}$.

The *suffix array* ${{\mathsf {s}}}{{\mathsf {a}}}[1,n]$ associated to ${\mathsf {s}}_{}$ is the permutation of [1, *n*] giving the lexicographic order of ${\mathsf {s}}_{}$’s suffixes, that is, for $i=1,\ldots ,n-1$, ${\mathsf {s}}_{}[{{\mathsf {s}}}{{\mathsf {a}}}[i],n] \prec {\mathsf {s}}_{}[{{\mathsf {s}}}{{\mathsf {a}}}[i+1],n]$.

The *longest common prefix* array $\mathsf {lcp} [1,n+1]$ is defined for $i=2,\ldots ,n$ by

1 $$\begin{aligned} \mathsf {lcp} [i]=\mathsf {LCP}({\mathsf {s}}_{}[{{\mathsf {s}}}{{\mathsf {a}}}[i-1],n],{\mathsf {s}}_{}[{{\mathsf {s}}}{{\mathsf {a}}}[i],n]); \end{aligned}$$

the $\mathsf {lcp}$ array stores the length of the longest common prefix (LCP) between lexicographically consecutive suffixes. For convenience we define $\mathsf {lcp} [1]=\mathsf {lcp} [n+1] = -1$.

**Fig. 1:**
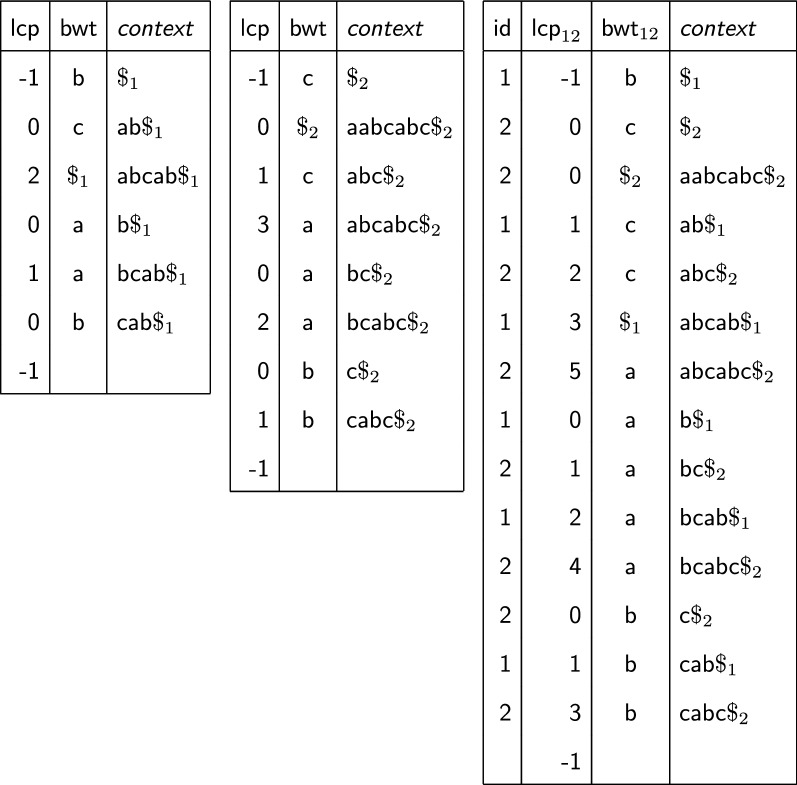
LCP array and BWT for ${\mathsf {s}}_{1}=\mathsf{abcab}\$_1$ and ${\mathsf {s}}_{2}=\mathsf{aabcabc}\$_{2}$, and multi-string BWT and corresponding LCP array for the same strings. Column id shows, for each entry of $\mathsf {bwt}_{12} = \mathsf{bc}\$_{2}\mathsf{cc}\$_1\mathsf{aaaabbb}$ whether it comes from ${\mathsf {s}}_{1}$ or ${\mathsf {s}}_{2}$

Let $${\mathsf {s}}_1[1,{n_1}],\ldots ,{\mathsf {s}}_k[1,{n_k}]$$ denote a collection of strings such that $${\mathsf {s}}_1[{n_1}] = \$_1,\ldots ,{\mathsf {s}}_k[{n_k}]=\$_k$$, where $$$_{1} < {\ldots }<$$ $$$_{k}$$ are *k* symbols not appearing elsewhere in $${\mathsf {s}}_1,\ldots ,{\mathsf {s}}_k$$ and smaller than any other symbol. Let $${{\mathsf {s}}}{{\mathsf {a}}}_{1\cdots k}[1,n]$$ denote the suffix array of the concatenation $${\mathsf {s}}_1\cdots {\mathsf {s}}_k$$ of total length $$n=\Sigma _{h=1}^{k}n_h$$. The *multi-string* BWT [19, 31] of $${\mathsf {s}}_1,\ldots ,{\mathsf {s}}_k$$, denoted by $$\mathsf {bwt}_{1\cdots k}[1,n]$$, is defined as

2 $$\begin{aligned} \mathsf {bwt}_{1\cdots k}[i] = {\left\{ \begin{array}{ll} {\mathsf {s}}_j[{\mathsf {n}}_{j}] &{} \text{ if } {{\mathsf {s}}}{{\mathsf {a}}}_{1\cdots k}[i] = \Sigma _{h=1}^{j-1}n_h + 1\\ {\mathsf {s}}_j[{{\mathsf {s}}}{{\mathsf {a}}}_{1\cdots k}[i]-\Sigma _{h=1}^{j-1}n_h - 1] &{} \text{ if } \Sigma _{h=1}^{j-1}n_h+1 < {{\mathsf {s}}}{{\mathsf {a}}}_{1\cdots k}[i] \le \Sigma _{h=1}^{j}n_h. \end{array}\right. } \end{aligned}$$

Essentially $\mathsf {bwt}_{1\cdots k}$ is a permutation of the symbols in ${\mathsf {s}}_1,\ldots ,{\mathsf {s}}_k$ such that the position in $\mathsf {bwt}_{1\cdots k}$ of ${\mathsf {s}}_{i}[j]$ is given by the lexicographic rank of its context ${\mathsf {s}}_{i}[j+1,n_i]$ (or ${\mathsf {s}}_{i}[1,n_i]$ if $j=n_i$). Figure 1 shows an example with $k=2$. Notice that for $k=1$, this is the usual Burrows–Wheeler Transform [1].

Given the suffix array $${{\mathsf {s}}}{{\mathsf {a}}}_{1\cdots k}[1,n]$$ of the concatenation $${\mathsf {s}}_1\cdots {\mathsf {s}}_k$$, we consider the corresponding LCP array $$\mathsf {lcp}_{1\cdots k}[1,n]$$ defined as in (1) (see again Fig. 1). Note that, for $$i=2,\ldots ,n$$, $$\mathsf {lcp}_{1\cdots k}[i]$$ gives the length of the longest common prefix between the contexts of $$\mathsf {bwt}_{1\cdots k}[i]$$ and $$\mathsf {bwt}_{1\cdots k}[i-1]$$. We stress that all practical implementations use a single $ symbol as end-marker for all strings to avoid alphabet explosion, but end-markers from different strings are then sorted as described, i.e., on the basis of the index of the strings they belong to.

### Computing multi-string BWTs

The gSACA-K algorithm [32], based on algorithm SACA-K [33], computes the suffix array for a string collection. Given a collection of strings of total length *n*, gSACA-K computes the suffix array for their concatenation in *O*(*n*) time using $(\sigma +1) \log n$ additional bits (in practice, only 2KB are used for ASCII alphabets). gSACA-K is time and space optimal for alphabets of constant size $\sigma =O(1)$. The *multi-string* $\mathsf {bwt}_{1\cdots k}$ of ${\mathsf {s}}_1,\ldots ,{\mathsf {s}}_k$ can be easily obtained from the suffix array as in (2). gSACA-K can also compute the $\mathsf {lcp}$ array $\mathsf {lcp}_{1\cdots k}$ still in linear time using only the additional space for the $\mathsf {lcp}$ values.

### Merging multi-string BWTs

The Gap algorithm [34], based on an earlier algorithm by Holt and McMillan [35], is a simple procedure for merging multi-string BWTs. In its original formulation the Gap algorithm can also merge LCP arrays, but in this paper we compute LCP values using a different approach more suitable for external memory execution. We describe here only the main idea behind Gap and refer the reader to [34] for further details.

For simplicity in the following we assume we are merging *k* single-string BWTs $\mathsf {bwt}_1=\mathsf {bwt} ({\mathsf {s}}_1),\ldots ,\mathsf {bwt}_{k}=\mathsf {bwt} ({\mathsf {s}}_k)$; the algorithm does not change in the general case where the inputs are multi-string BWTs. Computing $\mathsf {bwt}_{1\cdots k}$ amounts to sorting the symbols of $\mathsf {bwt}_1,\ldots ,\mathsf {bwt}_{k}$ according to the lexicographic order of their contexts, where the context of symbol $\mathsf {bwt}_{j}[i]$ is ${\mathsf {s}}_{j}[{{\mathsf {s}}}{{\mathsf {a}}}_j[i],n_j]$. By construction, the symbols in each $\mathsf {bwt} _j$ are already sorted by context, hence to compute $\mathsf {bwt}_{1\cdots k}$ we only need to merge $\mathsf {bwt}_1,\ldots ,\mathsf {bwt}_{k}$ without changing the relative order of the symbols within the input sequences.

The Gap algorithm works in successive iterations. During the *h*-th iteration it computes a vector $Z^{(h)}$ specifying how the entries of $\mathsf {bwt}_1,\ldots ,\mathsf {bwt}_{k}$ should be merged to have them sorted according to the first *h* symbols of their context. Formally, for $j=1,\ldots ,k$ the vector $Z^{(h)}$ contains $|\mathsf {bwt} _j|$ copies of the value *j* arranged so that the following property holds.

#### **Property 1**

*For* ${j_{1}},{j_{2}}\in \{1,\ldots ,k\}$, *the* $i_1$-*th occurrence of* $j_1$ *precedes the* $i_2$-*th occurrence of* $j_2$ *in* $Z^{(h)}$ *if and only if the length-h* *context of* $\mathsf {bwt} _{j_1}[i_1]$ *is lexicographically smaller than the length-h* *context of *$\mathsf {bwt} _{j_2}[i_2]$, *or the two contexts are equal and* $j_1 < j_2$. $\square$

Property 1 is equivalent to state that we can logically partition $Z^{(h)}$ into ${b(h)}+1$ blocks

3 $$\begin{aligned} Z^{(h)}[1,\ell _1],\; Z^{(h)}[\ell _1+1, \ell _2],\; \ldots ,\; Z^{(h)}[\ell _{b(h)}+1,n] \end{aligned}$$

such that each block corresponds to the set of symbols in $\mathsf {bwt}_{1\cdots k}$, whose contexts are prefixed by the same length-*h* string. The context of any symbol in block $Z^{(h)}[\ell _j+1, \ell _{j+1}]$ is lexicographically smaller than the context of the symbols in block $Z^{(h)}[\ell _k+1, \ell _{k+1}]$ with $k>j$; within each block, if ${j_{1}}<{j_{2}}$ the symbols of $\mathsf {bwt}_{{j_{1}}}$ precede those of $\mathsf {bwt}_{{j_{2}}}$. We keep explicit track of such blocks using a bit array $B[1,n+1]$ such that at the end of iteration *h* it is $B[i]\ne 0$ if and only if a block of $Z^{(h)}$ starts at position *i*. By Property 1, when all entries in *B* are nonzero, $Z^{(h)}$ describes how the $\mathsf {bwt}_{j}$ ($j=1,\ldots ,k$) should be merged to get $\mathsf {bwt}_{1\cdots k}$.

### Related approaches

The strategy used by Gap to build multi-string BWTs follows the idea, introduced by [35, 36], of merging partial BWTs, i.e. BWTs of subsets of the input collection. Interestingly, both previous algorithms for computing the BWT and LCP in external memory [19, 20] are also based on a merging strategy but instead of merging partial BWTs, they merge the arrays $L_1$, $L_2$, $L_3$, …, where $L_i$ consists of the symbols which are at distance *i* from the end of their respective strings. The symbols inside each $L_i$ are sorted as usual by context. In the example of Fig. 1, we would have $L_1 = \mathsf{bc}$ (since ${\mathsf {s}}_{1}$ ends with $\mathsf{b}\$_1$ and ${\mathsf {s}}_{2}$ ends with $\mathsf{c}\$_{2}$), $L_2 = \mathsf{ab}$, (since ${\mathsf {s}}_{1}$ ends with $\mathsf{ab}\$_1$ and ${\mathsf {s}}_{2}$ ends with $\mathsf{bc}\$_{2}$), $L_3 = \mathsf{ca}$ and so on. Note that in $L_3$ $\mathsf{c}$ precedes $\mathsf{a}$ since $\mathsf{c}$’s context $\mathsf{ab\$_1}$ is lexicographically smaller than $\mathsf{a}$’s context $\mathsf{bc\$_{2}}$. Clearly, merging the arrays $L_i$ yields the desired multi-string BWT and the authors of [19, 20] show how to compute also the LCP array. The algorithms in [19, 20] differ in how the merging is done: [19] uses a refinement of a technique introduced in [9, 10], while [20] uses a refinement of Holt and McMillan merging strategy [35, 36].

## The eGap algorithm

The eGap algorithm for computing the multi-string BWT and LCP array in external memory works in three phases. First it builds multi-string BWTs for sub-collections in internal memory, then it merges these BWTs in external memory and generates the LCP values. Finally, it sorts the LCP values in external memory.

### Phase 1: BWT computation

Given a collection of sequences ${\mathsf {s}}_{1}, {\mathsf {s}}_{2}, \ldots , {\mathsf {s}}_{k}$, we split it into sub-collections sufficiently small that we can compute the multi-string SA for each one of them in internal memory using the gSACA-K algorithm. After computing each SA, we compute the corresponding multi-string BWT and write it to disk in uncompressed form using one byte per character.

### Phase 2: BWT merging and LCP computation

This part is based on the Gap algorithm previously described but suitably modified to work efficiently in external memory (or in semi-external memory if there are at least *n* bytes of RAM). In the following we assume that the input consists of *k* BWTs $\mathsf {bwt} _1,\ldots ,\mathsf {bwt} _k$ of total length *n* over an alphabet of size $\sigma$. The input BWTs are read from disk and never moved to internal memory.

**Fig. 2:**
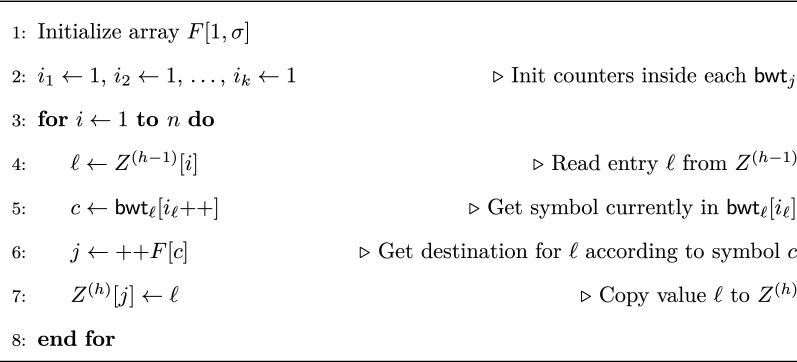
Outline of Gap’s main loop computing $Z^{(h)}$ from $Z^{(h-1)}$. Array *F* is initialized so that *F*[*c*] contains the number of occurrences of symbols smaller than *c* in $\mathsf {bwt}_{1\cdots k}$

The algorithm initially sets $Z^{(0)} = {\mathbf {1}}^{{n_1}}{\mathbf {2}}^{n_2}\ldots {\mathbf {k}}^{n_k}$ and $B = {\mathbf {1}}{\mathbf {0}}^{n-1} {\mathbf {1}}$. Since the context of every symbol is prefixed by the same length-0 string (the empty string), initially there is a single block containing all symbols. At iteration *h* the algorithm computes $Z^{(h)}$ from $Z^{(h-1)}$ as follows (see also Fig. 2). We define an array $F[1,\sigma ]$ such that *F*[*c*] contains the number of occurrences of characters smaller than *c* in $\mathsf {bwt}_{1\cdots k}$. *F* partitions $Z^{(h)}$ into $\sigma$ buckets, one for each symbol. Using $Z^{(h-1)}$ we scan the partially merged BWT, and whenever we encounter the BWT character *c* coming from $\mathsf {bwt} _\ell$, with $\ell \in \{1,\ldots ,k\}$, we store it in the next free position of bucket *c* in $Z^{(h)}$; note that *c* is not actually moved, instead we write $\ell$ in its corresponding position in $Z^{(h)}$. In our implementation, instead of using distinct arrays $Z^{(0)}, Z^{(1)}, \ldots$ we only use two arrays $Z^\mathsf {old}$ and $Z^{\mathsf {new}}$, that are kept on disk. At the beginning of iteration *h* it is $Z^\mathsf {old}= Z^{(h-1)}$ and $Z^{\mathsf {new}}= Z^{(h-2)}$; at the end $Z^{\mathsf {new}}= Z^{(h)}$ and the roles of the two files are swapped. While $Z^\mathsf {old}$ is accessed sequentially, $Z^{\mathsf {new}}$ is updated sequentially within each bucket, that is within each set of positions corresponding to a given character. Since the boundary of each bucket is known in advance we logically split the $Z^{\mathsf {new}}$ file in buckets and write to each one sequentially.

eGap computes LCP values exploiting the bitvector *B* used by Gap to mark the beginning of blocks (see Eq. 3) within each $Z^{(h)}$ (for simplicity the computation of *B* is not shown in Fig. 2). We observe that if *B*[*i*] is set to ${\mathbf {1}}$ during iteration *h* then $\mathsf {lcp}_{1\cdots k}[i] = h-1$, since the algorithm has determined that the contexts of $\mathsf {bwt}_{1\cdots k}[i]$ and $\mathsf {bwt}_{1\cdots k}[i-1]$ have a common prefix of length exactly $h-1$. We introduce an additional bit array $B_{x}[1,n+1]$ such that, at the beginning of iteration *h*, $B_{x}[i]={\mathbf {1}}$ iff *B*[*i*] has been set to ${\mathbf {1}}$ at iteration $h-2$ or earlier. During iteration *h*, if $B[i]={\mathbf {1}}$ we look at $B_{x}[i]$. If $B_{x}[i]={\mathbf {0}}$ then we know that *B*[*i*] has been set at iteration $h-1$: thus we output to a temporary file $F_{h-2}$ the pair $\langle i,h-2\rangle$ to record that $\mathsf {lcp}_{1\cdots k}[i]=h-2$, and we set $B_{x}[i]={\mathbf {1}}$ so no pair for position *i* will be produced in the following iterations. At the end of iteration *h*, file $F_{h-2}$ contains all pairs $\langle i,\mathsf {lcp}_{1\cdots k}[i]\rangle$ with $\mathsf {lcp} [i]=h-2$; the pairs are written in increasing order of their first component, since *B* and $B_{x}$ are scanned sequentially. These temporary files will be merged in Phase 3 to produce the LCP array.

As proven in [34, Lemma 7], if at iteration *h* of the Gap algorithm we set $B[i]={\mathbf {1}}$, then at any iteration $g \ge h+2$ processing the entry $Z^{(g)}[i]$ will not change the arrays $Z^{(g+1)}$ and *B*. Since the roles of the $Z^\mathsf {old}$ and $Z^{\mathsf {new}}$ files are swapped at each iteration, and at iteration *h* we scan $Z^\mathsf {old}= Z^{(h-1)}$ to update $Z^{\mathsf {new}}$ from $Z^{(h-2)}$ to $Z^{(h)}$, we can compute only the entries $Z^{(h)}[j]$ that are different from $Z^{(h-2)}[j]$. In particular, any range $[\ell ,m]$ such that $B_{x}[\ell ] = B_{x}[\ell +1] = \cdots = B_{x}[m] = {\mathbf {1}}$ can be added to a set of *irrelevant* ranges that the algorithm may skip in successive iterations (irrelevant ranges are defined in terms of the array $B_{x}$ as opposed to the array *B*, since before skipping an irrelevant range we need to update both $Z^\mathsf {old}$ and $Z^{\mathsf {new}}$). We read from one file the ranges to be skipped at the current iteration and simultaneously write to another file the ranges to be skipped at the next iteration (note that irrelevant ranges are created and consumed sequentially). Since skipping a single irrelevant range takes ${\mathcal {O}}(k+\sigma )$ time, an irrelevant range is stored only if its size is larger than a given threshold *t* and we merge consecutive irrelevant ranges whenever possible. In our experiments we used $t=\max (256,k+\sigma )$. In the worst case the space for storing irrelevant ranges could be ${\mathcal {O}}(n)$ but in actual experiments it was always less than 0.1*n* bytes.

As in the Gap algorithm, when all entries in *B* are nonzero, $Z^\mathsf {old}$ describes how the BWTs $\mathsf {bwt}_{j}$ ($j=1,\ldots ,k$) should be merged to get $\mathsf {bwt}_{1\cdots k}$, and a final sequential scan of the input BWTs along with $Z^\mathsf {old}$ allows to write $\mathsf {bwt}_{1\cdots k}$ to disk, in sequential order. Our implementation can merge at most $2^7 = 128$ BWTs at a time because we use 7 bits to store each entry of $Z^\mathsf {old}$ and $Z^{\mathsf {new}}$. These arrays are maintained on disk in two separate files; the additional bit of each byte are used to keep the current and the next copy of *B*. The bit array $B_{x}$ is stored separately in a file of size *n*/8 bytes. To merge a set of $k>128$ we split the input in subsets of cardinality 128 and merge them in successive rounds. In practice, the algorithm merges the multi-string BWTs produced by Phase 1. In our experiments the maximum number of sub-collections was 21.

*Semi-external version* We have also implemented a semi-external version of the merge algorithm that uses *n* bytes of RAM. The *i*-th byte is used to store $Z^\mathsf {old}[i]$ and $Z^{\mathsf {new}}[i]$ (3 bits each), *B*[*i*] and $B_{x}[i]$. This version can sort at most $2^3 = 8$ BWTs simultaneously; to sort *k* BWTs it performs $\log _8 k$ merging rounds. Although performing more rounds is clearly more expensive, this version stores in RAM all the arrays that are modified and reads from disk only the input BWTs and is therefore significantly faster.

### Phase 3: LCP merging

At the end of Phase 2 all $\mathsf {LCP}$-values have been written to the temporary files $F_h$ on disk as pairs $\langle i,\mathsf {lcp} [i]\rangle$. Each file $F_h$ contains all pairs with second component equal to *h* in order of increasing first component. The computation of the LCP array is completed using a standard external memory multiway merge [37, Chap. 5.4.1] of $\mathsf {maxlcp}$ sorted files, where $\mathsf {maxlcp}=\max _i(\mathsf {lcp}_{1\cdots k}[i])$ is the largest LCP value.

### Analysis

During Phase 1, gSACA-K computes the suffix array for a sub-collection of total length *m* using 9*m* bytes (8*m* bytes for ${{\mathsf {s}}}{{\mathsf {a}}}$ and 1*m* bytes for the text). If the available RAM is *M*, the input is split into subcollections of size $\approx M/9$. Since gSACA-K runs in linear time, if the input collection has total size *n*, Phase 1 takes ${\mathcal {O}}(n)$ time overall.

A single iteration of Phase 2 consists of a complete scan of $Z^{(h-1)}$ except for the irrelevant ranges. Since the algorithm requires $\mathsf {maxlcp}$ iterations, without skipping the irrelevant ranges the algorithm would require $\mathsf {maxlcp}$ sequential scans of ${\mathcal {O}}(n)$ items. Reasoning as in [34, Theorem 8] we get that by skipping irrelevant ranges the overall amount of data *directly* read/written by the algorithm is ${\mathcal {O}}( n\, \mathsf {avelcp})$ items where $\mathsf {avelcp}$ is the arithmetic average of the entries in the final LCP array. However, if we reason in terms of disk blocks, every time we skip an irrelevant range we discard the current block and load a new one (unless the beginning of the new relevant range is inside the same block; in that case or if the beginning of the new relevant range is in the block immediately following the current one, skipping the irrelevant range does not save any I/O). We can upper bound this extra cost, with an overhead of ${\mathcal {O}}(1)$ blocks for each irrelevant range skipped. Summing up, if the total number of skipped ranges is *Ir* and each disk block consists of ${\mathcal B}$ words, the I/O complexity of Phase 2 according to the theoretical model in [15] is ${\mathcal {O}}( Ir + n\, \mathsf {avelcp}/({{\mathcal {B}}}\log n))$ block I/Os (under the reasonable assumptions that the alphabet is constant, each entry in *Z* takes constant space, and we need a constant number of merge rounds). Although the experiments in “Experiments” section suggest that in practice *Ir* is small, for simplicity and uniformity with the previous literature we upper bound the cost of Phase 2 with ${\mathcal {O}}(n\, \mathsf {maxlcp})$ sequential I/Os (corresponding to ${\mathcal {O}}(n\, \mathsf {maxlcp}/({{\mathcal {B}}}\log n))$ block I/Os).

Phase 3 takes ${\mathcal {O}}(\lceil \log _\lambda \mathsf {maxlcp}\rceil )$ rounds; each round merges $\lambda$ LCP files by sequentially reading and writing ${\mathcal {O}}(n)$ bytes of data. The overall cost of Phase 3 is therefore ${\mathcal {O}}(n \log _\lambda \mathsf {maxlcp})$ sequential I/Os. In our experiments we used $\lambda =256$; since in our tests $\mathsf {maxlcp}< 2^{16}$ two merging rounds were always sufficient.

## Experiments

In this section we report on an experimental study comparing between the eGap algorithm and the other known external memory tools computing the BWT and LCP arrays of sequence collections. We implemented eGap in ANSI C based on the code of Gap [34] and gSACA-K [32]. eGap source code is freely available at https://github.com/felipelouza/egap/. All tested algorithms were compiled with GNU GCC ver. 4.6.3, with optimizing option -O3. The experiments were conducted on a machine with GNU/Linux Debian 7.0/64 bits operating system using an Intel i7-3770 3.4 GHz processor with 8 MB cache, 32 GB of RAM and a 2.0 TB SATA hard disk with 7200 RPM and 64 MB cache. The complete set of experiments took about 70 days of computing time.

**Table 1:** Datasets used in our experiments

Name	Size GB	N. of strings	Max Len	Ave Len	Max LCP	Ave LCP
short	8.0	85,899,345	100	100	99	27.90
long	8.0	28,633,115	300	300	299	90.28
pacbio.1000	8.0	8,589,934	1000	1000	876	18.05
pacbio	8.0	942,248	71,561	9116	3084	18.32

Columns 4 and 5 show the maximum and average lengths of the single strings. Columns 6 and 7 show the maximum and average LCPs of the collections

*Datasets* We used four real DNA datasets reported in Table 1 containing sequences of different lengths and structure. The sequences of the first three datasets were trimmed to make them of the same length, while the fourth dataset contains sequences of widely different lengths. short are Illumina reads from human genome (ftp://ftp.sra.ebi.ac.uk/vol1/ERA015/ERA015743/srf/). long are Illumina HiSeq 4000 paired-end RNA-seq reads from plant Setaria viridis (https://trace.ncbi.nlm.nih.gov/Traces/sra/?run=ERR1942989). pacbio.1000 and pacbio are PacBio RS II reads from Triticum aestivum (wheat) genome (https://trace.ncbi.nlm.nih.gov/Traces/sra/?run=SRR5816161). All datasets contain sequences over the A, C, G, T alphabet plus a string terminator symbol.

*Memory setting* To make a realistic external memory experimental setting one has to use an amount of RAM smaller than the size of the data. Indeed, if more RAM is available, even if the algorithm is supposedly not using it, the operating system will use it to temporarily store disk data and the algorithm will be no longer really working in external memory. This phenomenon will be apparent also from our experiments. For these reasons we reduced the available RAM to simulate three different scenarios: (i) input data 4 times larger than the available RAM, (ii) input data of approximately the same size as the RAM, and (iii) input data 4 times smaller than the RAM. We evaluated these scenarios with the complete 8 GB datasets from Table 1 (with 2 GB, 8 GB, and 32 GB RAM), and with the datasets trimmed to 1 GB (hence with 256 MB, 1 GB, and 4 GB RAM). The RAM was limited at boot time to a value equal to the amount assigned to the algorithm plus a small extra amount for the operating system (14 MB for the 256 MB instance and 64 MB for the others).

### Comparison with the existing algorithms

We compared eGap with the algorithm BCR [19] which is the current state of the art for BWT/LCP computation for collections of sequences. We used the bcr-lcp implementation from [38] since the previous implementation mentioned in [19] did not compute the LCP values correctly. We tested also the recently proposed algorithm bwt-lcp-em [20] using the code from [39]. As a reference we also tested the algorithm eGSA [14] using the code from [40]. eGSA computes the Suffix and LCP Arrays for collections of sequences in external memory: the disadvantage of this algorithm is that working with the Suffix Array could involve transferring to/from disk a much larger amount of data.

**Fig. 3:**
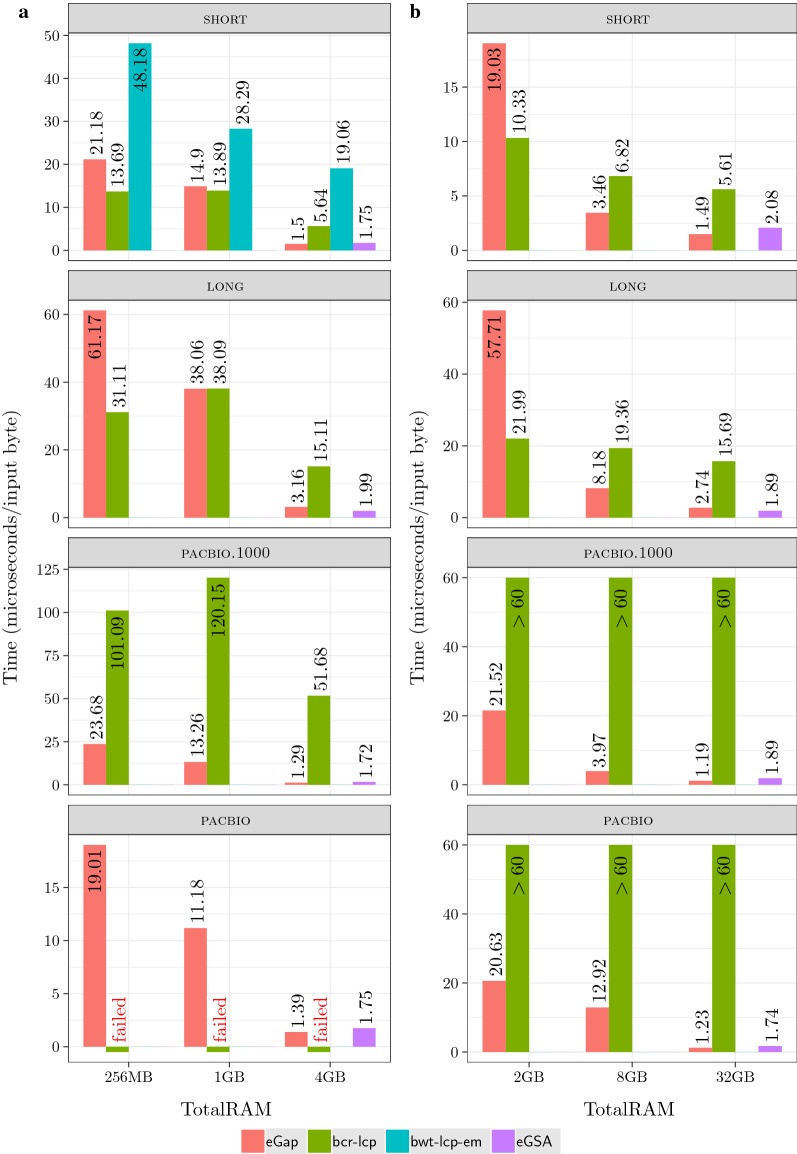
Running time in microseconds per input byte as a function of the available memory for the 1 GB datasets (left) and the 8 GB datasets (right)

*Limitations* We tested bwt-lcp-em only on the short 1 GB dataset since the implementation in [39] only supports collections of at most 2 GB and with strings of at most 253 symbols. We tested eGSA only with memory scenario (iii) (input data 4 times smaller than the RAM) since it was already observed in [14] that eGSA ’s running time degrades when the RAM is restricted to the input size. Finally, we could not test bcr-lcp on the pacbio 1 GB dataset since it stopped with an internal error after four days of computation. This is probably due to the presence of very long strings in the dataset since bcr-lcp was originally conceived for collections of short/medium length strings. The corresponding entries are marked as “failed” in Fig. 3. For the larger 8 GB datasets we stopped the experiments that did not complete after six days of CPU time, corresponding to 60 microseconds per input symbol. The corresponding entries are marked with “$>60$” in Fig. 3. Note that both bwt-lcp-em and bcr-lcp are active projects, so some of the limitations reported here could have been solved after our experiments were completed.

*Results:* The results of our experiments are summarized in Fig. 3. The bar plots on the left are for the 1 GB datasets showing the running time as function of the available RAM; the diagrams on the right are for the 8 GB datasets. The results show that for memory scenarios (i) and (ii) eGap and bcr-lcp have the better performance, whereas for scenario (iii) eGap and eGSA are the best options. The performance of bwt-lcp-em improves with the RAM size, but it is still 12 times slower than eGap for the short datasets with 4 GB of RAM.

The above results are in good accordance with the theoretical analysis. bcr-lcp complexity is ${\mathcal {O}}(n\, \mathsf {maxlen})$ sequential I/Os while eGap and bwt-lcp-em both take ${\mathcal {O}}(n\, \mathsf {maxlcp})$ sequential I/Os. For the short and long datasets the maximum length and the maximum LCP coincide and we see that when the available memory is only one fourth of the input size bcr-lcp is clearly the fastest option: indeed it is up to a factor 2.6 faster than eGap. This is no longer true when the available memory is equal or larger than the input size: in this case eGap is the fastest, probably because of its ability to exploit all the available memory using a semi-external strategy whenever possible. When the available memory is larger than the input size or for the pacbio.1000 dataset which has a very large $\mathsf {maxlen}$ then eGap is up to 40 times faster than bcr-lcp. Note that, in accordance with our heuristic analysis, eGap ’s running time per input byte appears to be roughly proportional to the *average* LCP of the collection. If we look at the datasets pacbio and pacbio.1000 we see that they have widely different maximum LCPs, yet their running times are very close similarly to their average LCPs.

Note that in the scenario (iii) eGSA is often the fastest algorithm and its running time appears to be less influenced by the size of the average or maximum LCP. Another advantage is that it also computes the Suffix Array, but it has the drawback of using a large amount of disk working space: 340 GB for a 8 GB input vs 56 GB used by eGap.

We conclude that, although eGap is not always the fastest algorithm, its running time is never too far from that of the best algorithm. In addition, eGap is the only algorithm that was able to complete all computations in all memory models. Although it was devised as an external memory algorithm, its ability to switch to a semi-external strategy if the memory is available makes it a very flexible tool. The comparison with the other algorithms in this setting is indeed not completely fair, since none of them is designed to take the available memory as a parameter in order to make the best use of it. Note that, as the available memory increases, all algorithms become faster because the operating system uses the RAM as a buffer but the speed improvement is different for different algorithms.

**Fig. 4:**
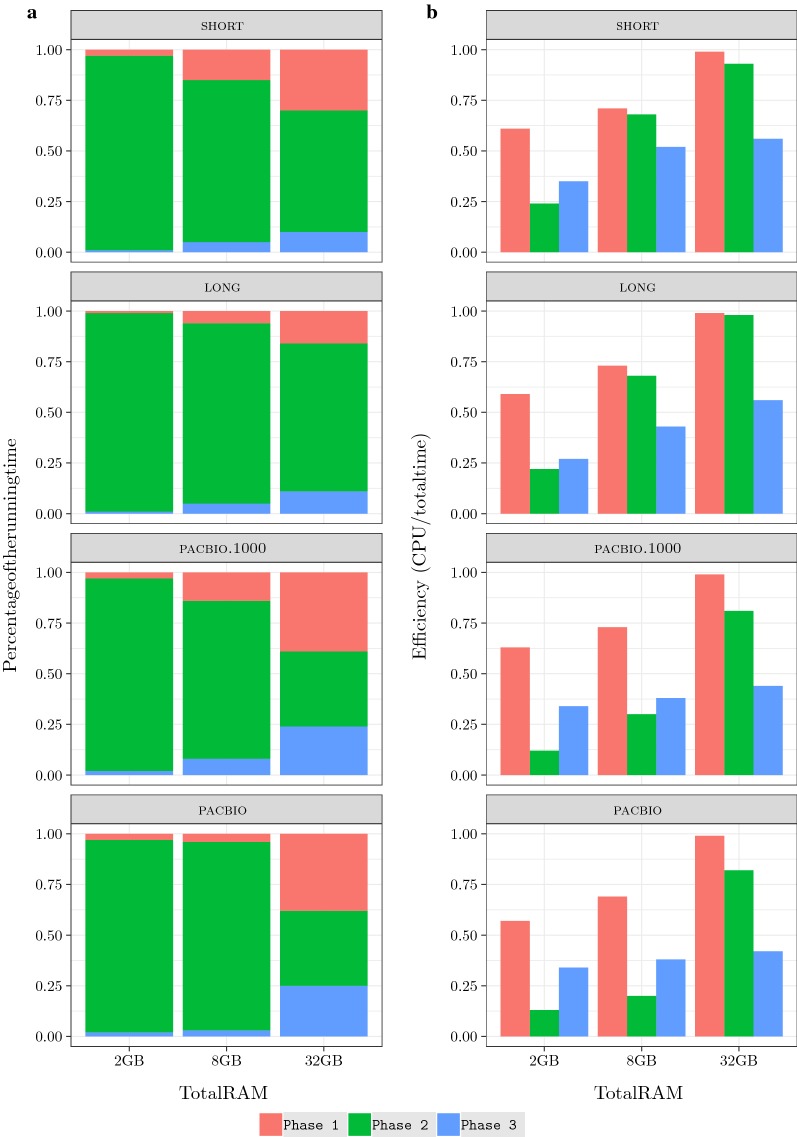
Running time in microseconds per input byte (left) and efficiency (right) for eGap ’s three phases

### Relative performance of eGap’s building blocks

We evaluated the percentage of time spent by each phase of eGap and their efficiency (percentage the CPU was busy) on the 8 GB datasets in the memory scenarios considered above, thus with RAM limited to (i) 1 GB, (ii) 8 GB, and (iii) 32 GB.

The results in Fig. 4 show that Phase 2 of eGap dominates the algorithm in general. The second phase took about $95\%$, $85\%$ and $50\%$ of the total time in scenarios (i), (ii), and (iii) respectively. If we look at the efficiency of the single phases, we see that they all improve with the RAM size. However, we notice that for any given memory scenario the efficiency of Phases 1 and 3 was almost the same for the different datasets, while Phase 2 has a different behavior. For the short and long datasets with 8 GB and 32 GB RAM, we see that Phase 2 efficiency is very close to Phase 1’s, while there is a sharp drop when using 2 GB RAM. For the pacbio datasets, the drop in Phase 2 efficiency is significant already when we use 8 GB RAM.

## Applications

In this section we show that the eGap algorithm, in addition to the BWT and LCP arrays, can output additional information useful to design efficient *external memory* algorithms for three well known problems on sequence collections: (i) the computation of maximal repeats, (ii) the all pairs suffix–prefix overlaps, and (iii) the construction of succinct de Bruijn graphs. For these problems we describe algorithms which are derived from known (internal memory) algorithms suitably modified so that they process the input data in a single sequential scan.

Our first observation is that eGap can also output the array which provides, for each $\mathsf {bwt}$ entry, the id of the sequence to which that entry belongs. In information retrieval this is usually called the Document Array, so in the following we will denote it by ${{\mathsf {d}}}{{\mathsf {a}}}$. In Phase 1 the gSACA-K algorithm can compute the ${{\mathsf {d}}}{{\mathsf {a}}}$ together with the $\mathsf {lcp}$ and $\mathsf {bwt}$ using only additional 4*n* bytes of space to store the ${{\mathsf {d}}}{{\mathsf {a}}}$ entries. These partial ${{\mathsf {d}}}{{\mathsf {a}}}$ ’s can be merged in Phase 2 using the $Z^{\mathsf {new}}$ array in the same way as the BWT entries. In the following we use $\mathsf {bwt}$, $\mathsf {lcp}$, and ${{\mathsf {d}}}{{\mathsf {a}}}$ to denote the multistring BWT, LCP and Document Array of a collection of *m* sequences of total length *n*. We write ${\mathsf {s}}$ to denote the concatenation ${\mathsf {s}}_{1} \cdots {\mathsf {s}}_{m}$ and ${{\mathsf {s}}}{{\mathsf {a}}}$ to denote the suffix array of ${\mathsf {s}}$. We will use ${\mathsf {s}}$ and ${{\mathsf {s}}}{{\mathsf {a}}}$ to describe and prove the correctness of our algorithms, but neither ${\mathsf {s}}$ nor ${{\mathsf {s}}}{{\mathsf {a}}}$ are used in the computations.

### Computation of maximal repeats

Different notions of maximal repeats have been used in the bioinformatics literature to model different notions of repetitive structure (see for example [21, 22]). We use a notion of maximal repeat from [41, Chap. 7]: we say that a string $\alpha$ is a *Type 1 maximal repeat* if $\alpha$ occurs in the collection at least twice and every extension, i.e. $c\alpha$ or $\alpha c$ with $c\in \Sigma$, occurs fewer times. We consider also a more restrictive notion: we say that a string $\alpha$ is a *Type 2 maximal repeat* if $\alpha$ occurs in the collection at least twice and every extension of $\alpha$ occurs at most once.

To compute Type 1 maximal repeats the crucial observation is that there is a substring of length $\ell$ that prefixes ${{\mathsf {s}}}{{\mathsf {a}}}$ entries $j, j+1, \ldots , i$ (and no others) iff $\mathsf {lcp} [k] \ge \ell$ for $k=j+1,\ldots ,i$, and both $\mathsf {lcp} [j]$ and $\mathsf {lcp} [i+1]$ are smaller than $\ell$. To ensure that the repeat is Type 1 maximal, we also require that there exists $h \in [j+1,i]$ such that $\mathsf {lcp} [h]=\ell$ and that the substring $\mathsf {bwt} [j,i]$ contains at least two distinct characters.

Our algorithm consists of a single sequential scan of $\mathsf {bwt}$ and $\mathsf {lcp}$. During the scan, we maintain a stack containing pairs ${\langle j, \mathsf {lcp} [h] \rangle }$ with $j \le h$ such that if ${\langle j', \mathsf {lcp} [h'] \rangle }$ is below ${\langle j, \mathsf {lcp} [h] \rangle }$ then $j'<j$ and $\mathsf {lcp} [h']<\mathsf {lcp} [h]$. In addition, when the scanning reaches position *i*, for every entry ${\langle j, \mathsf {lcp} [h] \rangle }$ in the stack it is $\mathsf {lcp} [h]=\min _{j\le k < i}\mathsf {lcp} [k]$, that is, $\mathsf {lcp} [h]$ is the smallest value in the range $\mathsf {lcp} [j,i-1]$.

We maintain the stack as follows. When we reach position *i*, if the entry ${\langle j, \mathsf {lcp} [h] \rangle }$ at the top of the stack has $\mathsf {lcp} [h]<\mathsf {lcp} [i]$ we push ${\langle i, \mathsf {lcp} [i] \rangle }$ on the stack. If $\mathsf {lcp} [h]=\mathsf {lcp} [i]$ we do nothing. If $\mathsf {lcp} [h]>\mathsf {lcp} [i]$ we pop from the stack all entries ${\langle j, \mathsf {lcp} [h] \rangle }$ with $\mathsf {lcp} [h]> \mathsf {lcp} [i]$; if the removal leaves at the top of the stack an entry ${\langle j', \mathsf {lcp} [h'] \rangle }$ with $\mathsf {lcp} [h'] < \mathsf {lcp} [i]$ we push on the stack a new entry ${\langle {\hat{\jmath }}, \mathsf {lcp} [i] \rangle }$ where ${\hat{\jmath }}$ is the first component of the last entry just removed from the stack. Note that in any case when we have completed the processing of position *i* the entry at the top of the stack has second component equal to $\mathsf {lcp} [i]$, and for each stack entry ${\langle j, \mathsf {lcp} [h] \rangle }$ it is $\mathsf {lcp} [h]=\min _{j\le k \le i} \mathsf {lcp} [k]$ as claimed.

We now prove that if ${\langle j', \mathsf {lcp} [h'] \rangle }$ is immediately below ${\langle j, \mathsf {lcp} [h] \rangle }$ then $\mathsf {lcp} [j-1]=\mathsf {lcp} [h']$. As we observed above, if at step *i* we push ${\langle i, \mathsf {lcp} [i] \rangle }$ on the stack, the previous top entry has second component equal to $\mathsf {lcp} [i-1]$ so the property holds for the first insertion of an entry ${\langle i, \mathsf {lcp} [\cdot ] \rangle }$. During the following steps it is possible that ${\langle i, \mathsf {lcp} [x] \rangle }$ is removed and immediately reinserted as ${\langle i, \mathsf {lcp} [y] \rangle }$ (with $\mathsf {lcp} [y]<\mathsf {lcp} [x]$), but since the preceding stack element does not change, is still holds true that $\mathsf {lcp} [i-1]$ is equal to the second component of the preceding element. Note that, since $\mathsf {lcp}$ values on the stack are strictly increasing, we conclude that for each stack entry ${\langle j, \mathsf {lcp} [h] \rangle }$ it is $\mathsf {lcp} [j-1] < \mathsf {lcp} [h]$.

Our algorithm outputs Type 1 maximal repeats when elements are popped from the stack. At step $i+1$ we pop from the stack all entries ${\langle j, \mathsf {lcp} [h] \rangle }$ such that $\mathsf {lcp} [h] > \mathsf {lcp} [i+1]$. Recall that by construction $\mathsf {lcp} [h] = \min _{j \le k\le i} \mathsf {lcp} [k]$. In addition $\mathsf {lcp} [j-1] < \mathsf {lcp} [h]$ and $\mathsf {lcp} [i+1]<\mathsf {lcp} [h]$. Thus, to ensure that we have found a Type 1 maximal repeat we only need to check that $\mathsf {bwt} [j-1,i]$ contains at least two distinct characters. To efficiently check this latter condition, for each stack entry ${\langle j, \mathsf {lcp} [h] \rangle }$ we maintain a bit vector $b_j$ of size $\sigma$ keeping track of the distinct characters in the array $\mathsf {bwt}$ from position $j-1$ to the next stack entry, or to the last seen position for the entry at the top of the stack. When ${\langle j, \mathsf {lcp} [h] \rangle }$ is popped from the stack its bit vector is or-ed to the previous stack entry in constant time; if ${\langle j, \mathsf {lcp} [h] \rangle }$ is popped from the stack and immediately replaced with ${\langle j, \mathsf {lcp} [i] \rangle }$ its bit vector survives as it is (essentially because it is associated with an index, not with a stack entry). Clearly, maintaining the bit vector does not increase the asymptotic cost of the algorithm.

Since at each step we insert at most one entry on the stack, the overall cost of our algorithm is ${\mathcal {O}}(n)$ time. The algorithm uses a stack of size bounded by ${\mathcal {O}}(\mathsf {maxlcp})$ words. For most applications $\mathsf {maxlcp}\ll n$ so it should be feasible to keep the stack in RAM. However, since a stack can also be implemented in external memory in ${\mathcal {O}}(1)$ amortized time per operation [42], we can state the following result.

#### **Theorem 1**

*We can compute all Type 1 maximal repeats in* ${\mathcal {O}}(n)$ *time executing a single scan of the arrays* $\mathsf {bwt}$ *and* $\mathsf {lcp}$ *using* ${\mathcal {O}}(1)$ *words of RAM.* $\square$

To find Type 2 maximal repeats, we are interested in consecutive LCP entries $\mathsf {lcp} [j], \mathsf {lcp} [j+1], \ldots , \mathsf {lcp} [i], \mathsf {lcp} [i+1]$, such that $\mathsf {lcp} [j]< \mathsf {lcp} [j+1] = \mathsf {lcp} [j+2] = \cdots = \mathsf {lcp} [i] > \mathsf {lcp} [i+1].$ Indeed, this implies that for $h=j, \ldots , i$ all suffixes ${\mathsf {s}}[{{\mathsf {s}}}{{\mathsf {a}}}[h], n]$ are prefixed by the same string $\alpha$ of length $\mathsf {lcp} [j+1]$ and every extension $\alpha c$ occurs at most once. If this is the case, then $\alpha$ is a Type 2 maximal repeat if all characters in $\mathsf {bwt} [j,i]$ are distinct since this ensures that also every extension $c\alpha$ occurs at most once. In order to detect this situation, as we scan the $\mathsf {lcp}$ array we maintain a candidate pair ${\langle j+1, \mathsf {lcp} [j+1] \rangle }$ such that $j+1$ is the largest index seen so far for which $\mathsf {lcp} [j]<\mathsf {lcp} [j+1]$. When we establish a candidate at $j+1$ as above, we initialize to zero a bit vector *b* of size $\sigma$ setting to **1** only entries $\mathsf {bwt} [j]$ and $\mathsf {bwt} [j+1]$. As long as the following values $\mathsf {lcp} [j+2], \mathsf {lcp} [j+3], \ldots$ are equal to $\mathsf {lcp} [j+1]$ we go on updating *b* and if the same position is marked twice we discard ${\langle j+1, \mathsf {lcp} [j+1] \rangle }$. If we reach an index $i+1$ such that $\mathsf {lcp} [i+1]>\mathsf {lcp} [j+1]$, we update the candidate to ${\langle i+1, \mathsf {lcp} [i+1] \rangle }$ and reinitialize *b*. If we reach $i+1$ such that $\mathsf {lcp} [i+1]<\mathsf {lcp} [j+1]$ and ${\langle j+1, \mathsf {lcp} [j+1] \rangle }$ has not been discarded, then a repeat of Type 2 (with $i-j+1$ repetitions) has been located.

#### **Theorem 2**

*We can compute all Type 2 maximal repeats in* ${\mathcal {O}}(n)$ *time executing a single scan of the arrays* $\mathsf {bwt}$ *and* $\mathsf {lcp}$ *using* ${\mathcal {O}}(1)$ *words of RAM.* $\square$

Note that when our algorithms discover Type 1 or Type 2 maximal repeats we know the repeat length and the number of occurrences so one can easily filter out non-interesting repeats (too short or too frequent). In some applications, for example the MUMmer tool [43], one is interested in repeats that occur in at least *r* distinct input sequences, maybe exactly once for each sequence. Since for these applications the number of input sequences is relatively small, we can handle these requirements by simply scanning the ${{\mathsf {d}}}{{\mathsf {a}}}$ array simultaneously with the $\mathsf {lcp}$ and $\mathsf {bwt}$ arrays and keeping track of the sequences associated to a maximal repeat using a bit vector (or a union-find structure) as we do with characters in the $\mathsf {bwt}$.

### All pairs suffix–prefix overlaps

In this problem we want to compute, for each pair of sequences ${\mathsf {s}}_{i}$ ${\mathsf {s}}_{j}$, the longest overlap between a suffix of ${\mathsf {s}}_{i}$ and a prefix of ${\mathsf {s}}_{j}$. Our solution is inspired by the algorithm in [24] which in turn was derived by an earlier Suffix-tree based algorithm [23]. The algorithm in [24] solves the problem using a Generalized Enhanced Suffix array (consisting of the arrays ${{\mathsf {s}}}{{\mathsf {a}}}$, $\mathsf {lcp}$, and ${{\mathsf {d}}}{{\mathsf {a}}}$) in ${\mathcal {O}}(n+m^2)$ time, which is optimal since *n* is the size of the input and there are $m^2$ longest overlaps. However, for large collections it is natural to consider the problem of reporting only the overlaps larger than a given threshold $\tau$ still spending ${\mathcal {O}}(n)$ time plus constant time per reported overlap. Our algorithm solves this more challenging problem.

In the following we say that a suffix starting at $${{\mathsf {s}}}{{\mathsf {a}}}[i]$$ is *special* iff it is a prefix of the suffix starting at $${{\mathsf {s}}}{{\mathsf {a}}}[i+1]$$, not considering the end-marker. This is equivalent to state that $${\mathsf {s}}[{{\mathsf {s}}}{{\mathsf {a}}}[i]+\mathsf {lcp} [i+1]]={\$}$$. For example, in Fig. 1 (right) the special suffixes are $$\mathsf{ab}\$_1$$, $$\mathsf{abc}\$_{2}$$, $$\mathsf{abcab}\$_1$$ $$\mathsf{b}\$_1$$, $$\mathsf{bc}\$_2$$, $$\mathsf{bcab}\$_1$$, $$\mathsf{c}\$_2$$, $$\mathsf{cab}\$_1$$. Notice that a special suffix starting at $${{\mathsf {s}}}{{\mathsf {a}}}[i]$$ has the form* v*$ with $$|v|= \mathsf {lcp} [i+1]$$; clearly only if $${{\mathsf {s}}}{{\mathsf {a}}}[i]$$ is special then *v* can be a suffix–prefix overlap. Note also that any suffix $${\$}$$ is always trivially special.

To efficiently solve the suffix–prefix overlaps problem, we modify Phase 2 of our algorithm so that it outputs also the bit array $\mathsf {xlcp}$ such that $\mathsf {xlcp} [i]={\mathbf {1}}$ iff the suffix starting at ${{\mathsf {s}}}{{\mathsf {a}}}[i]$ is special. To this end, we maintain an additional length-*n* bit array *S* such that, at the end of iteration *h*, $S[i] = \mathbf{1}$ if and only if the suffix starting at ${{\mathsf {s}}}{{\mathsf {a}}}[i]$ is special and it has length less than *h*, again not considering the end-marker symbol. The array *S* is initialized at the end of iteration $h=1$ as $S = \mathbf{1}^k\mathbf{0}^{n-k}$, consistently with the fact that in the final suffix array the first *k* contexts are strings consisting of just an end-marker, that are special suffixes and the only suffixes of length 0.

During iteration *h*, we update *S* as follows. With reference to the code in Fig. 2, whenever we use entry $Z^{(h-1)}[i]$ to compute $Z^{(h)}[j]$ for some *j*, if $S[i]=\mathbf{1}$ and $B[j+1]=\mathbf{0}$ then we set $S[j]=\mathbf{1}$.

#### **Lemma 1**

*The above procedure correctly updates the array* *S*.

#### *Proof*

We prove by induction that at the end of iteration *h*: (1) $S[i]=\mathbf{1}$ iff the suffix starting at ${{\mathsf {s}}}{{\mathsf {a}}}[i]$ is special and has length less than *h*, and (2) if $S[i]=\mathbf{1}$ the length-*h* context currently in position *i* is in the correct lexicographic position with respect to the final suffix array ordering (in other words, it is a prefix for ${\mathsf {s}}[{{\mathsf {s}}}{{\mathsf {a}}}[i],n]$).

For $$h=1$$ the result is true by construction. During iteration $$h>1$$, if we reach a position *i* such that $$S[i]=\mathbf{1}$$, then by inductive hypothesis the context in position *i* has the form * v*$ with $$|v| \le h-2$$. If *c* is the symbol we read at Step 5 of Fig. 2, then the context corresponding to position *j* is* cv*$. Since the context contains the end-marker, *j* is the correct lexicographic position of* cv*$ which is therefore the suffix corresponding to $${{\mathsf {s}}}{{\mathsf {a}}}[j]$$. If $$B[j+1]=\mathbf{0}$$, then $$\mathsf {lcp} [j+1]\ge h-1$$. Since $$\mathsf {lcp} [j+1] \le |c v| \le h-1$$, it follows that $$|cv|=\mathsf {lcp} [j+1]=h-1$$ and *S*[*j*] is special as claimed.

On the other hand, if at the end of iteration *h* it is $S[j]=\mathbf{0}$, then either it was $S[i]=\mathbf{0}$ or $B[j+1]=\mathbf{1}$ which implies $\mathsf {lcp} [j+1]< h-1$. In both cases the suffix starting at ${{\mathsf {s}}}{{\mathsf {a}}}[j]$ cannot be special and of length less than *h*. $\square$

Having established the properties of *S*, we can now show how to compute $\mathsf {xlcp}$. Recall that LCP values are computed as follows. In Phase 2, during iteration $h+1$ if $B[i+1]=\mathbf{1}$ and $B_{x}[i+1]=\mathbf{0}$ we output the pair $\langle i+1,h-1\rangle$ recording the fact that $\mathsf {lcp} [i+1]=h-1$. Such pairs are later sorted by their first component during Phase 3 to retrieve the LCP array. If ${{\mathsf {s}}}{{\mathsf {a}}}[i]$ is special, its corresponding suffix has length $\mathsf {lcp} [i+1]=h-1$ so, by the properties of *S*, at the beginning of iteration $h+1$ it is $S[i]=\mathbf{1}$. Thus, to compute $\mathsf {xlcp}$, instead of the pair $\langle i+1,h-1\rangle$ we output the triplet $\langle i+1,h-1,S[i]\rangle = \langle i+1,\mathsf {lcp} [i+1],\mathsf {xlcp} [i]\rangle$. After the merging is completed we sort the triplets by their first component and we derive both arrays $\mathsf {lcp}$ and $\mathsf {xlcp}$.

Our algorithm for computing the suffix–prefix overlaps longer than a threshold $\tau$, consists of a sequential scan of the arrays $\mathsf {bwt}$, $\mathsf {lcp}$, ${{\mathsf {d}}}{{\mathsf {a}}}$, and $\mathsf {xlcp}$. We maintain *m* distinct stacks, $\mathsf {stack}[1],\ldots ,\mathsf {stack}[m]$, one for each input sequence; $\mathsf {stack}[k]$ stores pairs $\langle j, \mathsf {lcp} [j+1]\rangle$ only if ${{\mathsf {s}}}{{\mathsf {a}}}[j]$ is a *special* suffix belonging to sequence *k* such that $\mathsf {lcp} [j+1]>\tau$. During the scan we maintain the invariant that for all stack entries ${\langle j, \mathsf {lcp} [j+1] \rangle }$, $\mathsf {lcp} [j+1]$ is the length of the longest common prefix (longer than $\tau$) between ${\mathsf {s}}[{{\mathsf {s}}}{{\mathsf {a}}}[j],n]$ and ${\mathsf {s}}[{{\mathsf {s}}}{{\mathsf {a}}}[i],n]$, where *i* is the position just scanned.

To maintain the invariant in amortized constant time per scanned position, we use the following additional structures:

- A stack $\mathsf {lcpStack}$ containing, in increasing order, the values $\ell$ such that some $\mathsf {stack}[k]$ contains an entry with LCP component equal to $\ell$;
- An array of lists $\mathsf {top}$ such that $\mathsf {top}[\ell ]$ contains the indexes *k* for which the entry at the top of $\mathsf {stack}[k]$ has LCP component equal to $\ell$;
- An array $\mathsf {daPtr}[1,m]$ such that $\mathsf {daPtr}[k]$ points to the entry *k* in the list $\mathsf {top}[\ell _k]$ containing it ($\mathsf {daPtr}[k]$ is used to remove such entry *k* from $\mathsf {top}[\ell _k]$ in constant time).

We maintain the above data structures as follows. When we reach position $i+1$ we remove all entries ${\langle j, \mathsf {lcp} [j+1] \rangle }$ such that $\mathsf {lcp} [j+1]>\mathsf {lcp} [i+1]$. We use $\mathsf {lcpStack}$ to determine which are the values $\ell$ such that some stack contains an entry $\langle j,\ell \rangle$ with $\ell >\mathsf {lcp} [i+1]$. For the value $\ell$ at the top of $\mathsf {lcpStack}$ we locate through $\mathsf {top}[\ell ]$ all stacks that contain an $\ell$-entry at the top. For each one of these stacks we remove the top entry $\langle j,\ell \rangle$ so that a new entry $\langle j',\ell '\rangle$, with $\ell ' <\ell$, becomes the new top of the stack. Then, if *k* is the stack that is being updated, we add *k* to $\mathsf {top}[\ell ']$, and a pointer to the new entry is saved in $\mathsf {daPtr}[k]$ (overwriting the previous pointer). When all entries of $\mathsf {top}[\ell ]$ have been processed, $\mathsf {top}[\ell ]$ is emptied and $\ell$ is popped from $\mathsf {lcpStack}$. The whole procedure is repeated until a value $\ell \le \mathsf {lcp} [i+1]$ is left at the top of $\mathsf {lcpStack}$.

Finally, if $\mathsf {xlcp} [i]={\mathbf {1}}$ and $\mathsf {lcp} [i+1]>\tau$, ${\langle i, \mathsf {lcp} [i+1] \rangle }$ is added to $\mathsf {stack}[{{\mathsf {d}}}{{\mathsf {a}}} [i]]$; this requires removing ${{\mathsf {d}}}{{\mathsf {a}}} [i]$ from the list $\mathsf {top}[\ell ]$ where $\ell$ is the previous top LCP value in $\mathsf {stack}[{{\mathsf {d}}}{{\mathsf {a}}} [i]]$; the position of ${{\mathsf {d}}}{{\mathsf {a}}} [i]$ in $\mathsf {top}[\ell ]$ is retrieved through $\mathsf {daPtr}[{{\mathsf {d}}}{{\mathsf {a}}} [i]]$. Also we add ${{\mathsf {d}}}{{\mathsf {a}}} [i]$ to $\mathsf {top}[\mathsf {lcp} [i+1]]$, and the pointer to this new element of $\mathsf {top}[\mathsf {lcp} [i+1]]$ is written to $\mathsf {daPtr}[{{\mathsf {d}}}{{\mathsf {a}}} [i]]$. Since the algorithm performs an amortized constant number of operations per entry ${\langle i, \mathsf {lcp} [i+1] \rangle }$, maintaining the above data structures takes ${\mathcal {O}}(n)$ time overall.

The computation of the overlaps is done as in [24]. When the scan reaches position *i*, we check whether $$\mathsf {bwt} [i]=$$$. If this is the case, then $${\mathsf {s}}[{{\mathsf {s}}}{{\mathsf {a}}}[i],n]$$ is prefixed by the whole sequence $${\mathsf {s}}_{{{\mathsf {d}}}{{\mathsf {a}}} [i]}$$, hence the longest overlap between a prefix of $${\mathsf {s}}_{{{\mathsf {d}}}{{\mathsf {a}}} [i]}$$ and a suffix of $${\mathsf {s}}_{k}$$ is given by the element currently at the top of $$\mathsf {stack}[k]$$, since by construction these stacks only contain special suffixes whose overlap with $${\mathsf {s}}[{{\mathsf {s}}}{{\mathsf {a}}}[i],n]$$ is larger than $$\tau$$. Note that using $$\mathsf {lcpStack}$$ and $$\mathsf {top}$$ we can directly access the stacks whose top element corresponds to an overlap with $${\mathsf {s}}_{{{\mathsf {d}}}{{\mathsf {a}}} [i]}$$ larger than $$\tau$$, hence the time spent in this phase is proportional to the number of reported overlaps. As in [24] some care is required to handle the case in which the whole string $${\mathsf {s}}_{{{\mathsf {d}}}{{\mathsf {a}}} [i]}$$ is a suffix of another sequence, but this can be done without increasing the overall complexity as in [24]. Since we spend constant time for reported overlap and amortized constant time for scanned position the overall cost of the algorithm, in addition to the scanning of the $$\mathsf {bwt}$$/$$\mathsf {lcp}$$/$$\mathsf {xlcp}$$/$${{\mathsf {d}}}{{\mathsf {a}}}$$ arrays, is $${\mathcal {O}}(n+E_\tau )$$, where $$E_\tau$$ is the number of suffix–prefix overlaps greater than $$\tau$$. Since all stacks can be implemented in external memory spending amortized constant time per operation, we only need to store in RAM $$\mathsf {top}$$ and $$\mathsf {daPtr}$$ that overall take $${\mathcal {O}}(m+\mathsf {maxlcp})$$ words.

#### **Theorem 3**

*Our algorithm computes all suffix–prefix overlaps longer than* $\tau$ *in time* ${\mathcal {O}}(n+E_\tau )$, *where* $E_\tau$ *is the number of reported overlaps, using* ${\mathcal {O}}(m+\mathsf {maxlcp})$ *words of RAM and executing a single scan of the arrays* $\mathsf {bwt}$, $\mathsf {lcp}$, ${{\mathsf {d}}}{{\mathsf {a}}}$ *and* $\mathsf {xlcp}$. $\square$

### Construction of succinct de Bruijn graphs

A recent remarkable application of compressed data structures is the design of efficiently navigable succinct representations of *de Bruijn graphs* [26–28]. Formally, a de Bruijn graph for a collection of strings consists of a set of vertices representing the distinct *k*-mers appearing in the collection, with a directed edge (*u*, *v*) iff there exists a $(k+1)$-mer $\alpha$ in the collection such that $\alpha [1,k]$ is the *k*-mer associated to *u* and $\alpha [2,k+1]$ is the *k*-mer associated to *v*.

The starting point of all de Bruijn graphs succinct representation is the BOSS representation [28], so called from the authors’ initials. For simplicity we now describe the BOSS representation of a *k*-order de Bruijn graph using the lexicographic order of *k*-mers, instead of the co-lexicographic order as in [28], which means we are building the graph with the direction of the arcs reversed. This is not a limitation since arcs can be traversed in both directions (or we can apply our construction to the input sequences reversed).

Consider the *N* *k*-mers appearing in the collection sorted in lexicographic order. For each *k*-mer $\alpha _i$ consider the array $C_i$ of distinct characters $c\in \Sigma \cup \{\$\}$ such that $c\alpha _i$ appears in the collection. The concatenation ${W}= C_1 C_2 \cdots C_N$ is the first component of the BOSS representation. The second component is a binary array $\mathsf {last}$, with $|\mathsf {last}| = |{W}|$, such that $\mathsf {last}[j]={\mathbf {1}}$ iff ${W}[j]$ is the last entry of some array $C_i$. Clearly, there is a bijection between entries in ${W}$ and graph edges; in the array $\mathsf {last}$ each sequence ${\mathbf {0}}^{i}{\mathbf {1}}$ ($i\ge 0$) corresponds to the outgoing edges of a single vertex with outdegree $i+1$. Finally, the third component is a binary array ${W}^{-}$, with $|{W}^{-}| = |{W}|$, such that ${W}^{-}[j]={\mathbf {1}}$ iff ${W}[j]$ comes from the array $C_i$, where $\alpha _i$ is the lexicographically smallest *k*-mer prefixed by $\alpha _i[1,k-1]$ and preceded by *W*[*j*] in the collection. This means that $\alpha _i$ is the lexicographically smallest *k*-mer with an outgoing edge reaching the node associated to *k*-mer $W[j]\alpha _i[1,k-1]$. Note that the number of ${\mathbf {1}}$’s in $\mathsf {last}$ and ${W}^{-}$ is exactly *N*, i.e. the number of nodes in the de Bruijn graph.

We now show how to compute ${W}$, $\mathsf {last}$ and ${W}^{-}$ by a sequential scan of the $\mathsf {bwt}$ and $\mathsf {lcp}$ array. The crucial observation is that the suffix array range prefixed by the same *k*-mer $\alpha _i$ is identified by a range $[b_i,e_i]$ of LCP values satisfying $\mathsf {lcp} [b_i]<k$, $\mathsf {lcp} [\ell ] \ge k$ for $\ell =b_i+1,\ldots ,e_i$ and $\mathsf {lcp} [e_i+1]<k$. Since *k*-mers are scanned in lexicographic order, by keeping track of the corresponding characters in the array $\mathsf {bwt} [b_i,e_i]$ we can build the array $C_i$ and consequently ${W}$ and $\mathsf {last}$. To compute ${W}^{-}$ we simply need to keep track also of suffix array ranges corresponding to $(k-1)$-mers. Every time we set an entry ${W}[j]=c$ we set ${W}^{-}[j]={\mathbf {1}}$ iff this is the first occurrence of *c* in the range corresponding to the current $(k-1)$-mers.

#### **Theorem 4**

*Our algorithm computes the BOSS representation of a de Bruijn graph in* ${\mathcal {O}}(n)$ *time using* ${\mathcal {O}}(1)$ *words of RAM, and executing a single scan of the arrays* $\mathsf {bwt}$ *and* $\mathsf {lcp}$. $\square$

If, in addition to the $\mathsf {bwt}$ and $\mathsf {lcp}$ arrays, we also scan the ${{\mathsf {d}}}{{\mathsf {a}}}$ array, then we can keep track of which sequences contain any given graph edge and therefore obtain a succinct representation of the colored de Bruijn graph [44]. Finally, we observe that if our only objective is to build the *k*-order de Bruijn graph, then we can stop the phase 2 of our algorithm after the *k*-th iteration. Indeed, we do not need to compute the exact values of LCP entries greater than *k*, and also we do not need the exact BWT but only the BWT characters sorted by their length *k* context.

## Conclusions

In this paper we have described eGap, a new algorithm for the computation of the BWT and LCP arrays of large collection of sequences. Depending on the amount of available memory, eGap uses an external or semi-external strategy for computing the BWT and LCP values. An experimental comparison of the available tools for BWT and LCP arrays computation shows that eGap is the fastest tool in many scenarios and was the only tool capable of completing the computation within a reasonable time frame for all kind of input data.

Another important feature of eGap is that, in addition to the BWT and LCP array, it can compute, without any asymptotic slowdown, two additional arrays that provide important information about the substrings of the input collection. We show how to use such information to design efficient external memory algorithms for three important problems for biosequences, namely the computation of maximal repeats, the computation of the all pairs suffix–prefix overlaps, and the construction of succinct de Bruijn graphs. Overall our results confirm the importance of the BWT and LCP arrays beyond their use for the construction of compressed full text indexes. This is in accordance with other recent results that have shown of they can be used directly to discover structural information on the underlying collection (see [45–47] and references therein).
